# Functional Identification of Salt-Stress-Related Genes Using the FOX Hunting System from *Ipomoea pes-caprae*

**DOI:** 10.3390/ijms19113446

**Published:** 2018-11-02

**Authors:** Mei Zhang, Hui Zhang, Jie-Xuan Zheng, Hui Mo, Kuai-Fei Xia, Shu-Guang Jian

**Affiliations:** 1 Key Laboratory of Applied Botany, South China Botanical Garden, Chinese Academy of Sciences, Guangzhou 510650, China; zhanghuir@mails.ucas.ac.cn (H.Z.); zhengjiexuan16@mails.ucas.ac.cn (J.-X.Z.); mohui@scbg.ac.cn (H.M.); xiakuaifei@scbg.ac.cn (K.-F.X.); 2 Key Laboratory of South China Agricultural Plant Molecular Analysis and Genetic Improvement, South China Botanical Garden, Chinese Academy of Sciences, Guangzhou 510650, China; 3 University of the Chinese Academy of Sciences, Beijing 100039, China

**Keywords:** salt stress, cDNA library, FOX gene hunting system, ROS accumulation, *Ipomoea pes-caprae* L.

## Abstract

*Ipomoea pes-caprae* is a seashore halophytic plant and is therefore a good model for studying the molecular mechanisms underlying salt and stress tolerance in plant research. Here, we performed Full-length cDNA Over-eXpressor (FOX) gene hunting with a functional screening of a cDNA library using a salt-sensitive yeast mutant strain to isolate the salt-stress-related genes of *I. pes-caprae* (*IpSR* genes). The library was screened for genes that complemented the salt defect of yeast mutant AXT3 and could grow in the presence of 75 mM NaCl. We obtained 38 candidate salt-stress-related full-length cDNA clones from the *I. pes-caprae* cDNA library. The genes are predicted to encode proteins involved in water deficit, reactive oxygen species (ROS) scavenging, cellular vesicle trafficking, metabolic enzymes, and signal transduction factors. When combined with the quantitative reverse transcription-polymerase chain reaction (qRT-PCR) analyses, several potential functional salt-tolerance-related genes were emphasized. This approach provides a rapid assay system for the large-scale screening of *I. pes-caprae* genes involved in the salt stress response and supports the identification of genes responsible for the molecular mechanisms of salt tolerance.

## 1. Introduction

Plants are continuously challenged by multiple stresses in nature and are also subjected to the evolutionary pressures imposed by natural selection. Among diverse adversity stresses, salinity is one of growing problems which affect plant growth and agricultural production, and even result in environmental degradation [1,2]. The mechanisms of salinity tolerance in plants have been classified into three types—ion exclusion, osmotic tolerance, and tissue tolerance [3]—or been summarized as osmotic stress and ionic stress [4]. During the various plant processes, several types of key genes are involved in the plant salinity tolerance, mainly by mediating Na^+^/K^+^ absorption, detoxification, and exclusion, and the functional genes mainly include channel proteins, transcription factors, or some metabolic enzymes or protective proteins [4,5].

Based on the adaptability of plants to high salinity, most plants are classified into two categories: glycophytes and halophytes. Compared with glycophytes, halophytes are defined as plants which can naturally survive in a saline environment and even prefer it [6]. Some halophytes that flourish in high-salinity conditions are termed as extremophiles [7]. In the course of evolution, halophytes or extremophiles have developed various adaptive mechanisms responding to salinity to ensure their survival and alternation of generations [8]. Research into halophyte stress-tolerance-related genes would improve the understanding of the adaptation of plants to environmental stress and provide a good foundation for formulating effective measures to modify the tolerance of crops to external stresses [1].

*Ipomoea pes-caprae* (Convolvulaceae) is a perennial herbaceous vine plant distributing mainly on sandy beaches or in sunny positions on the roadside in tropical and subtropical regions, and usually grows in a high-temperature, dry environment, exhibiting great advantages in salt tolerance and drought resistance [9,10]. As a typical halophyte plant with a high level of nutrient utilization efficiency, *I. pes-caprae* constitutes a superior wild plant resource and plays important roles in sand fixation, wind resistance, landscape greening, and ecological restoration of the vegetation in tropical and subtropical coral islands and coastal zones [9,11]. In addition, since *I. pes-caprae* is also a highly adversity-resistant perennial officinal plant with super excellent phenotypes of resistance to abiotic stresses, a molecular biology approach to identification of the stress-tolerant functional genes of this species to extreme environments has great potential for application and significance in plant science.

Salinity is a common abiotic stress that is usually associated with saline/alkali land or sea beaches, and salt accumulation in soil or water can inhibit plant growth and reduce absorption of water, thereby causing osmotic or water-deficit stress in vivo. In both earlier and more recent studies, much research has focused on the “omics” changes induced by external stresses [7,12]. However, due to the lack of genomic information for non-model plant species, the changes in transcriptomic, proteomic, or metabolomic processes caused by stress do not provide sufficient information for the cloning and isolation of stress-related genes in non-model plant species. The Full-length cDNA Over-eXpressor (FOX) gene hunting system is a functional screening method using the overexpression of full-length cDNA libraries in model plants (*Arabidopsis* or rice) or microorganisms (mainly yeast) [13,14,15,16,17], by which some unknown stress-related genes have been cloned and preliminarily identified. Actually, in much earlier studies, the functional screening work with yeast mutants was widely used to separate salt tolerance genes from plant cDNA libraries due to the lack of any genomic sequence information, even in model plants such as *Arabidopsis* [18,19], tobacco [20], or rice [21]. Since yeast (*Saccharomyces cerevisiae*) is a unicellular organism with the best research background, the functional screening of stress-tolerance-related genes in yeast with the FOX gene hunting system seems more simple and easier to operate than in model plants (such as *Arabidopsis* and rice). In recent years, this gene hunting system has also been widely applied to isolate salt tolerance genes in some extreme halophyte species without any genomic sequence information [15,16,22].

In the present study, we constructed a yeast expression cDNA library and performed functional screening work in order to identify the candidate genes encoding functional proteins involved in salt tolerance in *I. pes-caprae*. We obtained a preliminary series of candidate genes for increasing the salt tolerance of yeast that can be considered as salt-stress-related functional genes for further research.

## 2. Results

### 2.1. The Na^+^ and K^+^ Contents in I. pes-caprae

**Figure 1:**
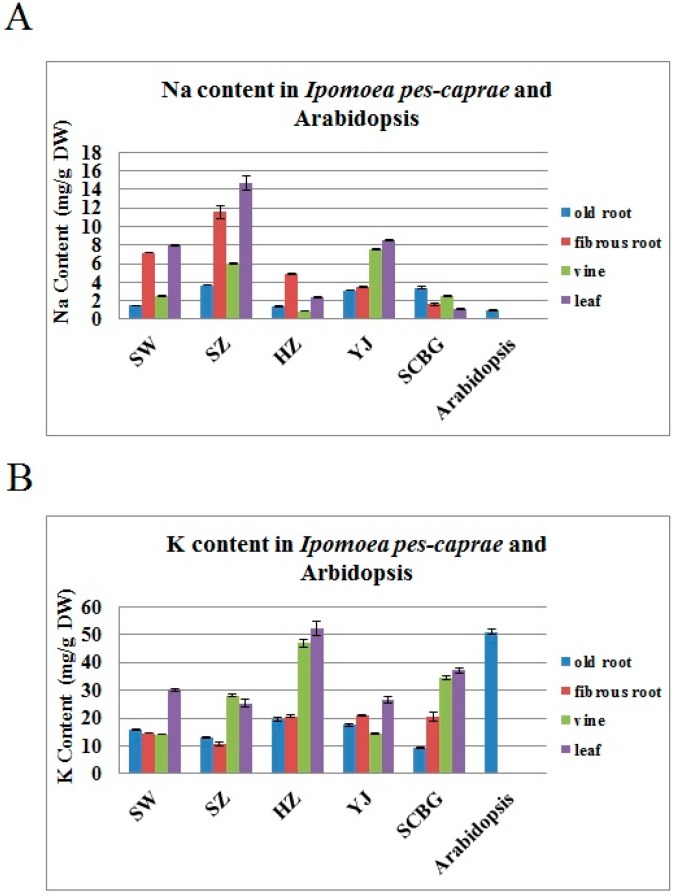
Na^+^ (**A**) and K^+^ (**B**) contents in *I. pes-caprae* collected from five different areas in Guangdong province. SW: sample from Shanwei (22°47′10.97″ N, 115°10′22.83″ E); SZ: sample from Shenzhen (22°32′13.16″ N, 114°29′11.26″ E); HZ: sample from Huizhou (22°41′24.81″ N, 114°44′48.02″ E); YJ: sample from Yangjiang (21°34′37.34″ N, 111°52′15.95″ E); SCBG: sample from South China Botanical Garden (23°18′49.89″ N, 113°35′84.32″ E). An Arabidopsis sample is also shown as a glycophyte control. All experiments were carried out for three replicates. The results shown are the mean ± SD (*n* = 3).

**Figure 2:**
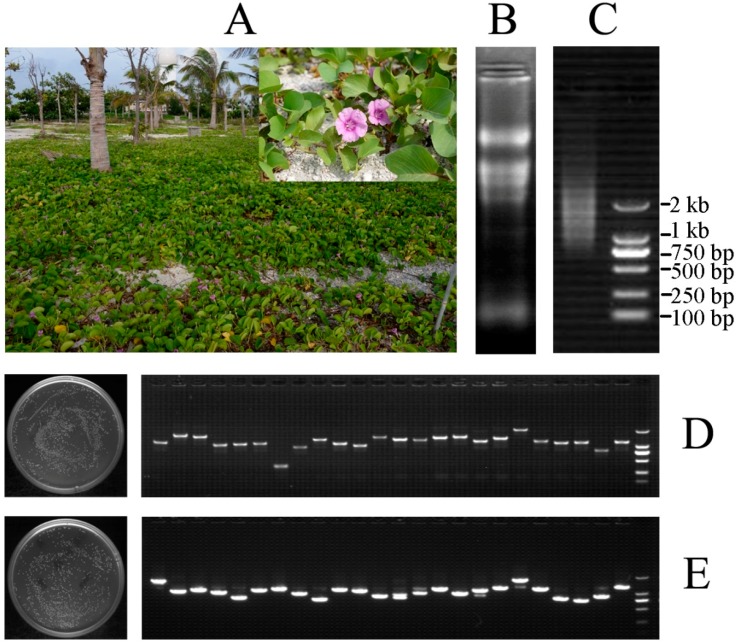
(**A**) The habitats of *I. pes-caprae*. This pictures were taken at the beach of Zhaoshu Island (16°58′ N, 112°16′ E) on 23 August 2016; (**B**) Analysis and detection of total RNA extracted from *I. pes-caprae* by 1% agarose gel electrophoresis; (**C**) Analysis and detection of mRNA purified from total RNA extracted from *I. pes-caprae* by 1% agarose gel electrophoresis; (**D**) Titer and inserted fragment detection of the entry cDNA library by colony PCR; (**E**) Titer and inserted fragment detection of the expression cDNA library by colony PCR.

Figure 1A shows that the concentration of Na^+^ in different tissues of *I. pes-caprae* collected from four different beaches (Shanwei, SW; Shenzhen, SZ; Huizhou, HZ; Yangjiang, YJ) and South China Botanical Garden (SCBG) in Guangdong province ranged from 1 to 16 mg g^−1^ dry weight. In general, the aerial parts of *I. pes-caprae* (leaves) living in beaches contained high amounts of Na^+^ compared with the underground portion (roots) (Figure 1A), while the SCBG sample did not show this salt-enriched feature. Although the Na^+^ content in the vines appeared to be lower than in the leaves or underground portion, if we consider the great biomass contribution from the vines, we can conclude that the aerial part of *I. pes-caprae* contained the bulk of the Na^+^ absorbed by the roots from the seawater (Figure 2A). As to the K^+^ content, all of the *I. pes-caprae* samples showed no obvious increase compared with *Arabidopsis* (Figure 1B), which indicated that the K^+^ might not be the cause of osmotic stress in *I. pes-caprae* plants in vivo.

Overall, compared with regular glycophytes such as *Arabidopsis* (Figure 1A), the Na^+^ contents in living plants of *I. pes-caprae* are much higher, which indicated that *I. pes-caprae* can accumulate a large amount of saline ions in vivo and has evolved a series of salt-tolerant mechanisms as an adaptive response to high-salt environments in order to lessen or even eliminate osmotic stress and the toxic ions from seawater. Additionally, osmotic stress damage is reduced by the waxy leaf surface and succulency. The vines of *I. pes-caprae* easily sprout adventitious roots, and the depth of primary roots can reach 3 meters, to make sure the plant gets water with little problem [23]. The above features indicate that *I. pes-caprae* can easily absorb water and limit the stomata transpiration, and thus possess strong drought tolerance under high temperature and strong light irradiation.

### 2.2. Construction of the cDNA Expression Library

An investigation of the habitat of *I. pes-caprae* revealed that it is a halophyte and is therefore associated with high salt tolerance (Figure 2A). For the purpose of isolating genes involved in salt tolerance in *I. pes-caprae*, a cDNA library was constructed with Gateway technology. Total RNA was separated from mixed samples of actively growing leaves, shoots, and roots of *I. pes-caprae*. The final amount of RNA was 842 μg with a concentration of 0.9351 μg μL^−1^ and A260/280 and A260/230 values for total RNA of 2.17 and 2.35, respectively. The total RNA had good quality with an RIN (RNA integrity number) value of 7.7. Subsequently, the mRNA was purified by magnetic beads and reached a final amount of 5 μg. In brief, by agarose gel electrophoresis, the total RNA and mRNA exhibited excellent quality for cDNA library construction (Figure 2B,C).

The cDNA entry library and pYES-DEST52 expression library were evaluated by counting the CFUs (colony-forming units). Generally, totals of 6.08 × 10^6^ and 5.48 × 10^6^ clones were contained in the entry library and expression library, respectively, which was sufficient to represent most of the genes expressed in *I. pes-caprae*. Meanwhile, the PCR insert fragment of 24 randomly picked clones from the entry library and expression library ranged from 0.5 to 3.0 kb with an average size over 1 kb (Figure 2D,E). Furthermore, 50 random clones from the pYES-DEST52 expression library were sequenced for full-length identification. After removing eight indistinguishable sequences, the ORFfinder program (available online: https://www.ncbi.nlm.nih.gov/orffinder/) was used to identify the longest open reading frames (ORFs) of the remaining 42 clones, and then the SmartBLAST analysis with the longest ORFs showed that some cDNAs were full-length and some were partial (Supplementary Data Table S1). Each possessed the ATG start codon (Supplementary Data Table S2), which indicated that the expression of cDNAs was guaranteed in the yeast and that the functional screening was effective. The sequences of these cDNA are listed in Supplementary Data Table S3.

### 2.3. Functional Screening of the cDNA Library for Mining the Candidate *IpSR* Genes

In this investigation, we chose a salt sensitive yeast strain, AXT3, to perform the FOX hunting assay of the *I. pes-caprae* cDNA library. AXT3 is functional knock-out with three Na^+^ transporter genes (*ena1-4*, *nha1*, and *nhx1*) and cannot grow well when the Na^+^ concentration in medium is above 50 mM [24,25]. From initial screening of the library plasmids, we chose 75 mM NaCl as a stringent selection concentration, with the empty expression vector pYES2 as a negative control. We found that under the challenge of 75 mM NaCl, the AXT3 yeast transformed with pYES2 could not grow into single clones on an SDG-Ura (supplied with adenine) plate, and a series of rescued single clones transformed with the library plasmids could be picked out and analyzed.

In brief, the plasmids (2–5 μg in total) of the pYES-DEST52 expression library were introduced into salt-sensitive AXT3 competent cells. A total of about 100 single yeast clones were obtained that could grow on NaCl-containing SDG-Ura medium (75 mM NaCl). These yeast clones were selected and amplified, and then the obtained plasmids were identified by alkali splitting and amplified in *E coli*. The single clone was selected and sequenced. After deleting some noncoding RNA sequences and poor-quality (including partials) or nonreferenced sequences, we ultimately acquired 38 meaningful reference recombinant clones encoding full-length cDNAs of *I. pes-caprae* genes. Finally, these 38 cDNAs were considered as candidate salt-stress-related genes in *I. pes-caprae* (*IpSRs*, Table 1). The sequences of these cDNA are listed in Supplementary Data Table S4.

### 2.4. Retransformation and Salt Tolerance Confirmation in Yeast

To avoid or prevent false positive results during the library screening process, and also to eliminate the possibility that one yeast clone might carry multiple genes, the plasmids carrying different *IpSRs* were reintroduced into the yeast strain AXT3 and its allele wild-type W303 to further confirm that these *IpSRs* could increase the salt tolerance of yeast.

**Figure 3:**
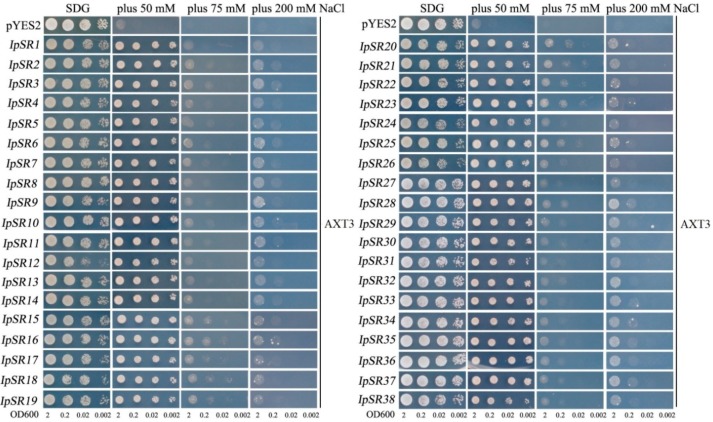
Salinity-tolerance confirmations in the yeast salt-sensitive mutant AXT3 of 38 clones (*IpSR1* to *38*) via library screening from *I. pes-caprae*. The yeast cultures (OD600 to 2) were serially diluted to OD600 values of 0.2, 0.02, and 0.002, and then 2 μL of yeast liquid was spotted onto SDG-Ura (supplied with adenine for AXT3) plates with or without NaCl (0, 50, 75, and 200 mM NaCl) for 5–10 days. The empty vector pYES2 represents the negative control.

As observed from Figure 3, all 38 genes showed increased salt tolerance in the yeast mutant strain AXT3 under different NaCl concentrations. At the 50 mM NaCl level, all 38 *IpSRs* almost recovered the salt sensitivity of AXT3, and all the transformant yeast clones showed recovered growth in comparison to the control AXT3 transformed with the empty vector, pYES2. Meanwhile, at higher NaCl levels (75 mM and 200 mM), the 38 *IpSRs* elevated the salt tolerance of AXT3 to some extent compared with the control AXT3.

**Figure 4:**
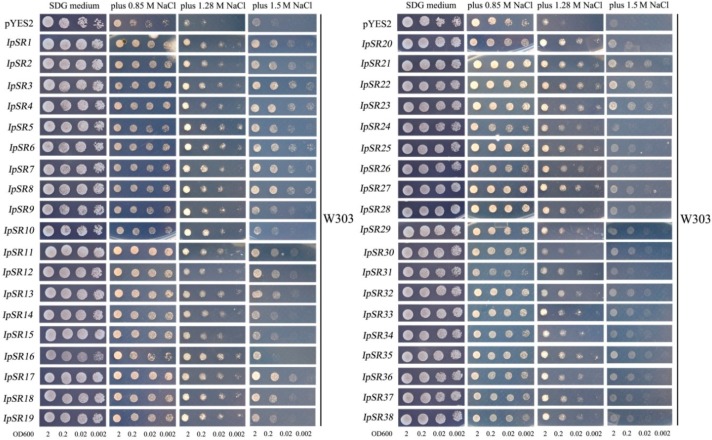
Salinity-tolerance confirmations in yeast wild-type strain W303 of 38 clones (*IpSR1* to *38*) via library screening from *I. pes-caprae*. The yeast culture (OD600 to 2) was serially diluted to OD600 values of 0.2, 0.02, and 0.002, and then 2 μL of yeast liquid was spotted onto SDG-Ura (supplied with adenine/leucine/histidine/tryptophan for W303) plates with (0.85, 1.28, and 1.5 M NaCl) or without NaCl for 5–10 days at 30 °C. The empty vector pYES2 represents negative control.

We also performed this assay in the wild-type yeast strain W303. As shown in Figure 4, the results showed that in the W303 carrying salt tolerance genes, the 38 *IpSRs* grew better than those with the empty vector pYES2 in the medium containing 0.85 M, 1.28 M, and 1.5 M NaCl, while no difference was observed in the growth of all yeast in the media lacking salt. These results further demonstrated the salt tolerance function of the 38 candidate genes from *I. pes-caprae* in yeast.

### 2.5. H_2_O_2_ Sensitivity Assays in Yeast

**Figure 5:**
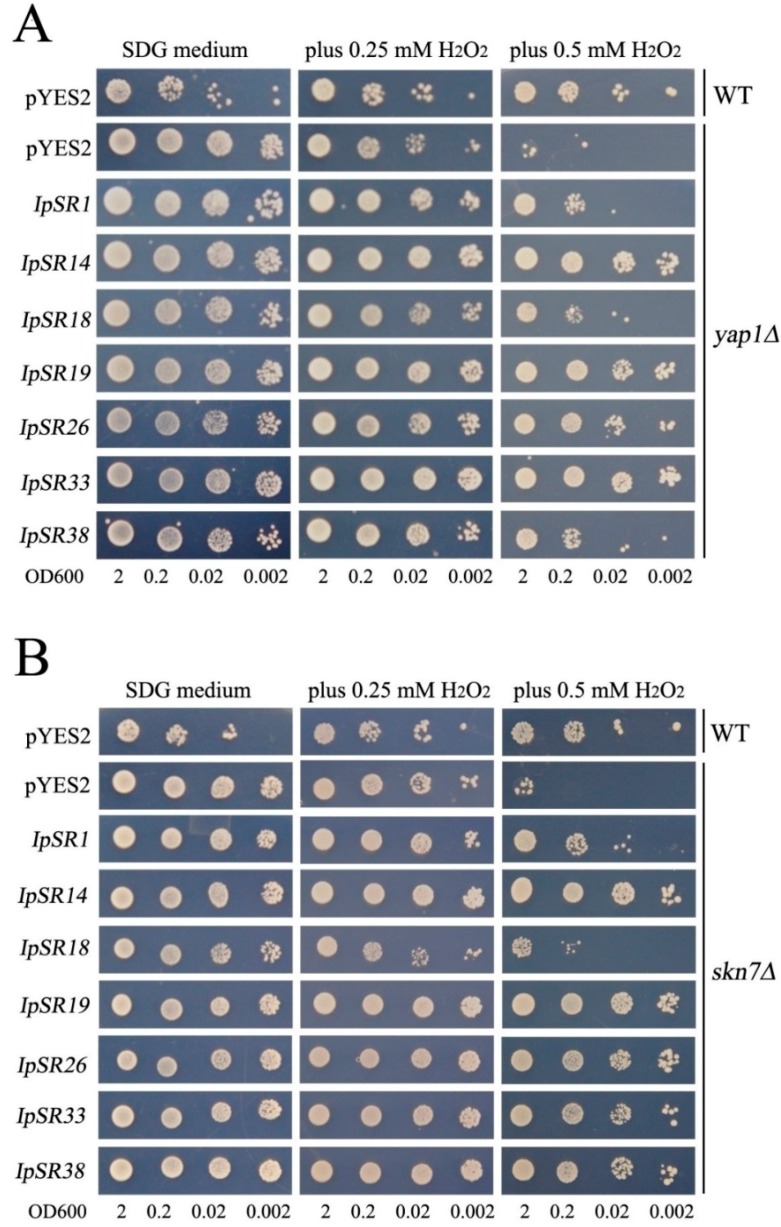
The oxidative resistance test of seven *IpSR* cDNAs (*IpSR1*, *IpSR14*, *IpSR18*, *IpSR19*, *IpSR26*, *IpSR33*, and *IpSR38*) overexpressed in the yeast mutant *yap1Δ* (**A**) and *skn7Δ* (**B**). The yeast culture (OD600 to 2) was serially diluted to OD600 values of 0.2, 0.02, and 0.002, and then 2 μL of yeast liquid was spotted onto SDG-Ura plates without or with H_2_O_2_ (0.25 mM and 0.5 mM) for 5–10 days at 30 °C. As a negative control, the mutant strain *yap1Δ* was transformed with the empty vector pYES2. As a positive control, wild-type yeast BY4741 (WT) was transformed with the empty vector pYES2.

In plants, salt challenge causes osmotic disruption, which in turn leads to ROS production [6]; therefore, oxidant stress pathways might be involved in the salt tolerance pathway. In our research, we assessed the antioxidative activities of some *IpSR* genes, including *IpSR1* (ABA/water stress/ripening-induced, *IpASR*), *IpSR14* (syntaxin/t-SNARE family protein, *IpSNARE*), *IpSR18* (catalase, *IpCAT*), *IpSR19* (stress responsive alpha-beta barrel domain protein, *IpSRP*), *IpSR26* (late embryogenesis abundant protein, *IpLEA*), *IpSR33* (glutathione S-transferase, *IpGST*), and *IpSR38* (dehydrin, *IpDHN*). Recombinant plasmids pYES-DEST52 carrying the above *IpSR* cDNAs were introduced into yeast H_2_O_2_-sensitive mutants *yap1Δ* and *skn7Δ*. The yeast genes *YAP1* and *SKN7* encode two transcriptional factors that play regulatory roles in oxidative stress [26], and mutations of these two genes will result in a decrease in oxidation resistance, exhibiting a sensitive phenotype to H_2_O_2_. Our results showed that the above-mentioned seven *IpSR* genes all elevated the tolerance to H_2_O_2_ of transformed yeast (*yap1Δ* and *skn7Δ*). This indicates that the products encoded by these *IpSR* genes all possess some antioxidative activities (Figure 5A,B).

### 2.6. qRT-PCR Analysis of Candidate Genes under Salt and Osmotic Stress

**Figure 6:**
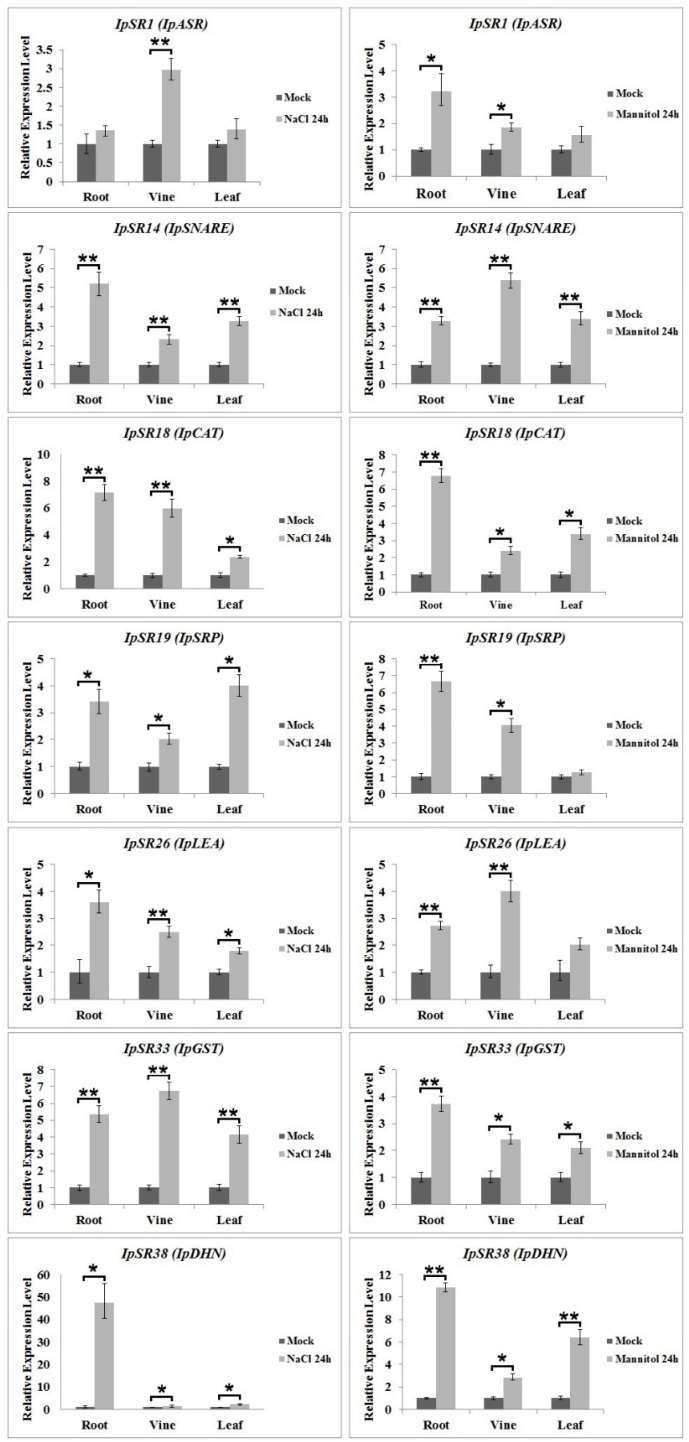
RT-PCR analyses of seven salinity-tolerant candidate genes (*IpSR1*, *IpSR14*, *IpSR18*, *IpSR19*, *IpSR26*, *IpSR33*, and *IpSR38*) in the roots, vines, and leaves of *I. pes-caprae* seedlings under salinity (left, 300 mM NaCl) and osmotic (right, 300 mM mannitol) stresses at 0 and 24 h. The house-keeping gene *IpUBQ* (GenBank accession number: MF502417) was used as a reference gene. All determinations were carried out for three biological replicates. The results shown are the mean ± SD (*n* ≥ 3). * and ** indicate significant differences in comparison with the wild type at 0.01 < *p* < 0.05 and *p* < 0.01, respectively (Student’s *t*-test).

In addition to the biochemical functions of the IpSR proteins expressed in yeast, the transcriptional changes of *IpSRs* in *I. pes-caprae* also reflect their biological roles under stress treatments. Therefore, we performed qRT-PCR analysis of 38 genes from desiccation- or osmotic-stressed *I. pes-caprae* (Figure 6; Supplementary Data Figures S1 and S2). We focused here on the induced expression of some *IpSRs*. In general, the seven *IpSR* genes whose products possess antioxidant activities in yeast, including *IpSR1*, *IpSR14*, *IpSR18*, *IpSR19*, *IpSR26*, *IpSR33*, and *IpSR38*, all without exception exhibited obvious characteristics of induced expression under both salt and osmotic stress (Figure 6). Furthermore, in the other 31 candidate *IpSRs*, eight genes showed significantly induced patterns under these two water imbalance stresses, including *IpSR2* (ATP synthase delta chain), *IpSR4* (stem-specific protein TSJT1-like protein), *IpSR8* (endoplasmic reticulum vesicle transporter), *IpSR11* (fructokinase), *IpSR16* (adenosylhomocysteinase 1), *IpSR24* (hypothetical protein AT3G52710), *IpSR25* (phosphomannomutase), and *IpSR27* (probable leucine-rich repeat receptor-like serine/threonine-protein kinase At1g56140). There were also three *IpSRs*, including *IpSR9* (stem-specific protein TSJT1-like), *IpSR20* (DNA-directed RNA polymerase II subunit RPB7), and *IpSR29* (phytosulfokines-like) that showed decreased expression patterns under both treatments. Some *IpSR* genes only showed obvious salt-inducible expression patterns (*IpSR10*, *IpSR12*, *IpSR15*, *IpSR23*, *IpSR32,* and *IpSR35*) or obvious osmotic-inducible expression patterns (*IpSR21* and *IpSR27*) (Supplementary Data Figures S1 and S2).

## 3. Discussion

Halophytes are considered to be the best germplasms for saline agriculture since they possess a strong capacity to thrive under extremely saline conditions [27]. Most crops are glycophytes; therefore, identifying the genes responsible for superior salinity tolerance in halophytes has great potential for the development of salinity-tolerant glycophytes, especially for the genetic modification of crops to improve salt tolerance. To characterize the genes involved in the salt stress response in *I. pes-caprae*, we screened the cDNA library of *I. pes-caprae* with the salt-sensitive yeast mutant AXT3, in which three Na^+^ transporter genes (*ena1-4*, *nha1*, and *nhx1*) were functionally deleted. Although we did not obtain any salt ion transporter genes during the screening process, we did still isolate some salt-stress-related candidate genes from *I. pes-caprae* (Table 1). This can be reduced to two reasons: firstly, the quality of the *I. pes-caprae* cDNA library is not good enough to isolate more functional key salt tolerance genes, or a broader screening is still needed to obtain more functional genes; secondly, as a single-cell organism, the yeast cannot fully reflect the salt tolerance process in multicellular plants and especially cannot simulate ion translocation or exclusion during our screening assay. In any case, further functional characterization of some key candidate genes conferring salt tolerance isolated from this assay will be helpful for deepening our understanding of the molecular mechanisms of the osmotic stress response and should provide some excellent and operative functional genes for genetic improvements for stress tolerance in crops in the future.

Halophytes can thrive under high salinity by adopting two strategies, namely, salt tolerance and salt avoidance [8]. Generally, euhalophytes possess strong salinity tolerance due to succulence, enabling the storage of more salty ions [28]. *Ipomoea pes-caprae* is a common pantropical creeping vine plant with slight leaf succulence and obvious stem (or vine) succulence. In our research, the Na^+^ content in the leaves and vines of *I. pes-caprae* showed apparently higher levels than in the roots (Figure 1A), indicating that *I. pes-caprae* growing in saline land can absorb and store salt in aboveground tissues. This differs from crinohalophytes and pseudohalophytes, in which the salts are secreted to the surroundings or stored in the roots or some cortical tissues of the aerial parts [29]. Here, we chose *I. pes-caprae* as a candidate for identifying salt-stress-related genes, and it was speculated that in *I. pes-caprae* the cells are severely challenged by salt stresses; thus, the multifunctionality of the salt tolerance genes will be more direct and distinctive.

Numerous studies have been done to provide a better understanding of the high salinity tolerance mechanisms of halophytes, with the purpose of isolating some key functional salt tolerance genes allowing halophytes to grow and flourish in high-saline environments and to breed crops with better performance under salinity stress [3,7,30]. However, some molecular biologists have assumed that only basic biochemical tolerance mechanisms, mainly related to water deficiency and osmotic stress processes, may be sufficient to confer a response to salt [31]. Even though only osmotic stress may still trigger many primary responses, including morphological and developmental changes, adjustment in ion transport, and metabolic changes, some secondary stress signal transduction should occur in turn [32]. It is obvious that in halophytes some molecular regulation and responding systems exist that differ from those in glycophytes and which are involved in maintaining regular growth and metabolism of the plant under salt stress.

In general, corresponding to the three categories of salt tolerance mechanisms in plants, the *HKT* (high-affinity potassium transporter) genes and the *SOS* (salt overly sensitive) pathway mainly mediate in regulating Na^+^ transport within ion exclusion processes in plants to limit the entry of salt via the roots; in plant tissue tolerance, vacuolar Na^+^/H^+^ antiporters (*NHX*) or V-PPase/V-ATPase play roles in controlling ion concentration and distribution, or enzymes for the biosynthesis of compatible solutes and proteins for the detoxification of ROS play roles to different extents in improving crop salinity tolerance. As to the third one, osmotic tolerance, although there are no specific candidate genes responding to it, several categories of functional genes, such as ROS generation and detoxification pathways, osmoregulation or ion homeostasis, salt-responsive genes, and transcription factors, even some chaperones, have been proved to be coordinately linked with this stress sensing and signal transduction [3,30]. From the holistic perspective, the osmotic stress as a consequence of ion and water imbalance in the whole plant might also have a greater effect on crop growth than the ionic toxicity, especially at low to moderate salinity levels [3,4].

In plant cells, many environmental stresses induce the accumulation of ROS, which then results in the expression of a series of stress response genes [33]. It is also known that oxidative stress signaling and ROS detoxification are both essential components of salinity stress tolerance mechanisms in halophytes [6]. In this research, we focused on the crosstalk between ROS detoxification and salt tolerance by assessing gene expression regulation by salt stress, and determined seven candidate genes that are involved in the salt stress response. Following the work of Roy et al. [3] and Mishra et al. [30], we classified all of these genes into the category responding to osmotic stress tolerance, mainly by mediating the ROS detoxification and ion/water homeostasis. Plant genetic engineering and manipulation of these novel salt-stress-related genes from *I. pes-caprae* may facilitate the development of glycophytes, particularly crops, with improved salt tolerance, and in the future may hold great promise in improving agricultural productivity under high salinity and drought stress. Several important candidate genes involved in salt tolerance isolated in this assay are listed and discussed in the following subsections.

### 3.1. Plant Abscisic Acid, Stress, and Ripening-Induced (ASR) Proteins

In this research, we obtained a yeast clone containing *IpASR* cDNA encoding an abscisic acid stress ripening-related protein, which showed high amino acid identities with the *Suaeda liaotungensis* ASR protein SlASR (AGZ20206.1) [34] and the *Salicornia brachiate* ASR protein SbASR-1 (ACI15208.1) [35]. ASR proteins play biochemical roles not only as transcription factors, but also as chaperones [36,37], and a number of reports have indicated that ASR members are involved in the salt and drought stress response which is gaining increasing attention due to water shortage issues in agriculture. Homology analysis of different plant ASR proteins indicated that IpASR could play similar biological roles in responding to high salt and drought stress. In our research, *IpASR* could partly complement the salt-sensitive defect of the yeast mutant strain AXT3 (Figure 3), and could also elevate the salt tolerance of the wild yeast strain W303 (Figure 4). Further experiments in yeast strains *yap1Δ* and *skn7Δ* suggested that *IpASR* could also improve oxidation resistance in yeast (Figure 5). The expression of *IpASR* in *I. pes-caprae* seedlings was also obviously induced under salt (300 mM NaCl) and osmotic stress (300 mM mannitol) challenges (Figure 6), which further indicated that *IpASR* might play pivotal roles in the adaptation of *I. pes-caprae* as a halophyte to extremely salty and dry environments.

### 3.2. LEA Proteins

Plant LEA proteins are hydrophilic and intrinsically disordered proteins which have been variously proposed to protect cellular structures from the effects of water loss by acting as a hydration buffer, by sequestering ions, by direct protection of other proteins or membranes, or by renaturing unfolded proteins [38]. Among them, dehydrins belong to group II LEA proteins, which are considered to be stress proteins involved in the formation of protective responses of plants to osmotic stress [39]. Many previous studies regarding the ectopic expression of individual *LEA* genes, including *dehydrins*, in various organisms have also provided support for functions in cellular protection under conditions of stress, including salinity, frost, and, in particular, drought [40,41]. In our study, yeast strains (AXT3 and W303) transformed with either a *LEA* gene (*IpLEA*) or a *dehydrin* gene (*IpDHN*) cloned from *I. pes-caprae* subjected to salinity stress exhibited better survival than those with the empty vectors (Figure 3 and Figure 4) and also exhibited an increased tolerance against the oxidant H_2_O_2_ in *yap1Δ* and *skn7Δ* (Figure 5), indicating that *IpLEA* and *IpDHN* could positively regulate salinity tolerance and mediate the response to oxidative stress. Our RT-PCR analysis showed obvious induced patterns under salt (300 mM NaCl) and osmotic stress (300 mM mannitol) challenges (Figure 6), suggesting possible cellular protective effects regulated by salinity and osmotic stress. The functions of *IpLEA* and *IpDHN* cloned from *I. pes-caprae* in regulating salinity tolerance deserve further consideration.

### 3.3. SNARE Proteins

Soluble *N*-ethylmaleimidesensitive fusion protein attachment protein receptors (SNARE) proteins constitute a type of membrane-anchored proteins that play key roles in vesicle-associated membrane fusion events during cellular vesicle trafficking between individual compartments of the endomembrane system, including exocytosis and endocytosis [42]. Considering that vesicle trafficking is a cellular housekeeping activity, SNARE may function in the spatial distribution of ion transporters that are important for plant development, as well as for ion detoxification caused by salt stress. Meanwhile, vesicle trafficking may also be influenced by the ion gradients they establish within the expanding cell [43]. Several reports have shown that over-expression of plant *SNARE* genes could improve the salt and drought tolerance of transgenic plants [44,45,46], which indicates that SNARE proteins might be involved in membrane stability, the K^+^/Na^+^ ratio, and antioxidant machinery [45]. NCBI SmartBlast analysis revealed that the IpSNARE (IpSR14) protein has a high sequence identity (47% amino acid identity under 77% query cover) to a SNARE-like superfamily protein from *A. thaliana* (NP_567842.1; At4g30240). In our research, we speculated that *IpSNARE* might be involved in Na^+^ and H_2_O_2_ redistribution in yeast by facilitating vesicle trafficking and improving the tolerance of yeast to salt and H_2_O_2_ (Figure 3, Figure 4 and Figure 5). The expression of *IpSNARE* was also induced by salt and osmotic stress in vivo (Figure 6). The results suggest that *IpSNARE* could be a potential candidate gene for increasing salinity and drought tolerance in crop plants for sustainable agriculture in arid and/or saline soil.

### 3.4. Catalase and Glutathione S-Transferase

Halophytic adaptation to salt stress involves a series of biochemical pathways and many active compounds including antioxidant enzymes. Catalase and glutathione S-transferase are two types of antioxidant enzymes responsible for ROS scavenging in plants [47]. Catalases can directly detoxify H_2_O_2_ generated from photorespiratory pathways, and also can effectively relieve the restrained growth caused by salt or other abiotic stresses [48]. Although there is little evidence for the up-regulated expression of catalase genes in plants for improving salt tolerance, some studies involving the knock-out or knock-down of catalase genes have indicated that they play significant roles against salinity in plants [49]. A catalase gene from the cyanobacterium *Anabaena*, *katB*, was strongly induced in response to osmotic stress or desiccation, and inactivation of *katB* resulted in enhanced sensitivity to salt stress in *Anabaena* cells [50]. Glutathione S-transferases (GSTs), as a type of detoxification enzyme, have versatile functions in multiple aspects of plant growth and development [51]. It has been confirmed that *GSTs* are induced by a variety of biotic and abiotic stresses [52]. Several plant glutathione transferase genes have been proved by plant transgenic assays mediating osmotic stress tolerance, mainly by ROS detoxification [53,54,55,56]. Here, we identified a catalase gene and a GST gene by functional screening with the *I. pes-caprae* cDNA library (Table 1), and there is little doubt that these two genes can improve the tolerance of yeast to H_2_O_2_ (Figure 5). RT-PCR analysis indicated that the expression patterns of the two genes challenged by salt and osmotic stress were both up-regulated (Figure 6), which further indicated that these two genes (*IpSR18* and *IpSR33*, defined as *IpCAT* and *IpGST*, respectively) might be involved in osmotic and water deficit stresses by mediating ROS equilibrium. Furthermore, our study also implied that *IpCAT* and *IpGST* may be potential candidate genes to be used in genetic engineering for enhancing abiotic stress tolerance.

### 3.5. Other Stress-Responsive Proteins

We also identified some other possible stress-responsive genes, including *IpSR3* (nudix hydrolase), *IpSR8* (endoplasmic reticulum vesicle transporter), *IpSR10* and *IpSR23* (proteasome inhibitors), *IpSR19* (stress responsive alpha-beta barrel domain protein), and *IpSR28* (dnaj protein-like protein) (Table 1). Since their homologous proteins in other species might mediate stress or hormone responses [57,58,59,60,61], future research on the functional identification of these genes should focus on their possible roles in abiotic stress. Of these, *IpSR19*, encoding a stress-responsive alpha-beta barrel domain protein, also named GLYCOLATE OXIDASE3 (UP3) in *Arabidopsis*, is a peroxisomal protein [62] that also showed oxidation resistance in yeast strains (Figure 5) and was up-regulated under salt and osmotic stress challenges (Figure 6). We proposed that the IpSR19 protein might also be involved in salt and drought tolerance by maintaining ROS equilibrium in *I. pes-caprae*.

To date, little genome information is available on *I. pes-caprae*, and no transcriptome and proteome data have been reported. Here, in this study, we adopted a cDNA library and screening strategy, the FOX hunting system, to obtain functional genes with full-length cDNAs responding to specific abiotic stresses within a short span of time. This approach may be particularly useful for the identification of some functional genes in wild plants lacking genetic information, and will be of great convenience for the elucidation of salt-tolerant or other stress-related genes. The FOX gene hunting approach was first proposed by Ichikawa T. et al. for the purpose of isolating functional genes in *Arabidopsis* that could result in a mutant phenotype via overexpression of the genes [63]. Subsequently, this technology has also been applied to rice with activation tags in a large generated rice population to identify useful traits and functional genes [14,64]. In *Eutrema salsugineum* (a halophyte, relative of *Arabidopsis*, also known as *Thellungiella salsuginea* and formerly *Thellungiella halophila*), two functional genes responding to salt or heat stress were also identified with the FOX hunting system [65,66]. Also, in another Brassicaceae halophytic relative of *Arabidopsis*, *Lepidium crassifolium*, the cDNA library was screened with the FOX hunting system in *Arbidopsis*, and the proteins GDSL-like lipase and Acyl CoA binding protein 6 were supposed to be involved in salt tolerance and osmotic stress tolerance, respectively [67]. After a decade of development, this strategy has been identified as a powerful tool for elucidating functional genomics and facilitating the discovery of useful genes in model plants or crops [17,68,69] or digging useful candidate genes from some wild plants [65,66,67]. The functional screening and identification of genes by transgenic assays in plants is a time-consuming and costly process during the first course of cDNA library screening. A modified FOX gene hunting system has been applied with a central change in the screening method, that is, with functional identification in yeast instead of in a plant; in this way, the experimental cycle of FOX hunting was greatly reduced [15,16,22]. For some wild plants lacking any genetic information, the FOX gene hunting approach with a novel gain-of-function strategy showed a greater advantage in the once-off and wide acquisition of full-length cDNAs of functional genes in a short time. In this research, a modified FOX gene hunting system was applied, resulting in the identification of 38 full-length cDNAs encoding candidate salt tolerance genes from *I. pes-caprae*.

In summary, in this paper we described a highly efficient approach for isolating salt tolerance genes from *I. pes-caprae* based on a FOX gene hunting system for the large-scale screening of functional genes. A high-quality cDNA expression library was successfully constructed and 38 potential *IpSRs* were identified. Several known genes involved in salt tolerance also showed oxidation resistance in yeast, and the transcriptional expression levels were detected. Interestingly, our results indicated that several pathways might participate in the regulation of salt tolerance in *I. pes-caprae*, including those related to water deficit, ROS scavenging, and cellular vesicle trafficking. These results provide the first insight into the salt tolerance gene network in *I. pes-caprae*. However, the biochemical and molecular mechanisms regarding the manner in which these novel genes regulate salinity tolerance are unknown. Further analysis of these *IpSRs* could provide deeper insights into the survival mechanisms of halophytes in extreme environments, and the identified genes could then serve as useful targets for the genetic modification of glycophytic plants for improved stress tolerance.

## 4. Materials and Methods

### 4.1. Plant Materials, Growth Conditions, and Stress Treatments

The seeds and young plants of *I. pes-caprae* were harvested from August to October around the seaside of Huizhou city (22°41′24.81″ N, 114°44′48.02″ E), Guangdong province. The young leaves and shoots were used to construct a cDNA library. For culturing the seedlings of *I. pes-caprae*, the seeds were sterilized with 70% ethanol followed by seed coat breaking with emery paper prior to placement onto MS basal salts distributed in plates with sand and soil collected outdoors from April to November in Guangzhou city. The seedlings were used for salt tolerance assays 30 days after germination. Subsequently, salt (300 mM NaCl) and osmotic (300 mM mannitol) stresses were applied to *I. pes-caprae* seedlings to detect the expression patterns of some candidate salt-tolerant genes. Experiments were performed following a completely randomized design with three replications and were repeated three times.

### 4.2. Measurement of Na^+^ and K^+^ Contents in I. pes-caprae

Whole plants of *I. pes-caprae* were harvested from four different beaches in Guangdong province, including Shenzhen (22°32′13.16″ N, 114°29′11.26″ E), Huizhou (22°41′24.81″ N, 114°44′48.02″ E), Yangjiang (21°34′37.34″ N, 111°52′15.95″ E), and Shanwei (22°47′10.97″ N, 115°10′22.83″ E); a sample series of *I. pes-caprae* growing in South China Botanical Garden (23°18′49.89″ N, 113°35′84.32″ E) cultivated with freshwater and regular soil was used as a control, as well as adult *Arabidopsis* (ecotype: col-0) plants cultured in a greenhouse under regular conditions. The different tissues of *I. pes-caprae* collected from those five areas were separated into fibrous roots, old roots, vines, and leaves, and then each sample (about 10 g) was cleaned with water and dried in a constant-temperature drying oven at 70 °C; the whole *Arabidopsis* plants were treated with the same protocol. Dried samples were crushed by a vibrating and ball-mining mixed machine (GSM 06, Siebtechnik, Mülheim, Germany). About 0.2 g of sample powder (accurate to 0.0001 g) was placed in a polytetrafluoroethylene tube and 6 mL of analytically pure concentrated nitric acid was added to each sample overnight at room temperature. The samples were then treated with a microwave digestion system according to the operating manual. Following microwave digestion, the sample liquids were transferred to new 50 mL volumetric flasks, diluted with ddH_2_O to a certain volume, and mixed. The contents of Na^+^ and K^+^ in the sample solutions were measured with a Perkin-Elmer inductively coupled plasma atomic absorption spectrometer. Each tissue sample was collected from three dependent adjacent plants in the same spot.

### 4.3. cDNA Library Construction and Quality Examination Assays

Total RNAs were extracted with TRIzol (Invitrogen, Thermo Fisher Scientific Inc., Waltham, MA, USA) and mRNAs were isolated from the total RNAs using the FastTrack^®^ MAG mRNA Isolation Kit (Invitrogen, Thermo Fisher Scientific Inc., Waltham, MA, USA). The integrity and purity of total RNAs extracted from the plants was detected by 1% agarose gel electrophoresis and a Nucleic Acid Analyzer (NanoDrop 2000, Thermo Fisher Scientific Inc., USA), and purified mRNAs were subjected to the same analysis. The total mRNAs were reverse-transcribed to the cDNAs using a SMART cDNA Synthesis Kit (Clontech, Takara Bio, Dalian, China) according to the manufacturer’s instructions. The cDNA libraries were constructed based on the Gateway^®^ cloning technology (Invitrogen, Thermo Fisher Scientific Inc., USA). In brief, the double strand-cDNAs were cloned into the pDONR222 vector using BP reaction (Gateway™ BP Clonase™ II Enzyme, Invitrogen), and the entry clone library was obtained. Through the LR reaction (Gateway™ LR Clonase™ II Enzyme mix, Invitrogen) of the Gateway^®^ cloning technology, the yeast expression vector pYES-DEST52 was ligated with the entry clone library to generate the secondary yeast expression library, in which the total *I. pes-caprae* cDNAs were inserted into the yeast expression cassette under the galactose-induced promoter pGAL1.

The mixtures of the BP and LR reactions containing an entry clone library and secondary expression library were introduced separately into competent cells of *E. coli* DH10B by electroporation. Thousand-fold diluted libraries were then cultured overnight on a solid medium plate (LB plus 50 mg L^−1^ kanamycin or 100 mg L^−1^ ampicillin) and the clones were counted. Twenty-four single clones of the two libraries were selected separately to obtain a monoclonal colony to detect the length of the inserted fragment of the libraries. The primer pairs for pDONR222 and pYES-DEST52 were pD22F/pD22R (5′-3′): GTAAAACGACGGCCAGTC/CCAGGAAACAGCTATGAC and T7/pY52R (5′-3′): TAATACGACTCACTATAGGG/AGGGTTAGGGATAGGCTTACC. Fifty random clones in the secondary expression library were sequenced; thereafter, the start codon (ATG) was found following BLASTX analysis.

### 4.4. Yeast Mutant Strains and Functional Screening

The salt-sensitive yeast mutant strain AXT3 (*ena1-4Δ::HIS3*; *nha1Δ::LEU2*; *nhx1Δ::TRP1*) and its wild-type strain W303 (*MATa*; *his3-11_15*; *leu2-3_112*; *ura3-1*; *trp1Δ2*; *ade2-1*; *can1-100*) were kindly provided by Pardo [24] and Jiang [25]. H_2_O_2_-sensitive mutant yeast strains *yap1Δ* (Y00569, BY4741; *MATa*; *ura3Δ0*; *leu2Δ0*; *his3Δ1*; *met15Δ0*; *YML007w::kanMX4*), *skn7Δ* (Y02900, BY4741; *MATa*; *ura3Δ0*; *leu2Δ0*; *his3Δ1*; *met15Δ0*; *YHR206w::kanMX4*) and their isogenic wild-type (WT) BY4741 (Y00000, *MATa*; *his3Δ1*; *leu2Δ0*; *met15Δ0*; *ura3Δ0*) were all obtained from Euroscarf (available online: http://www.euroscarf.de/index.php?name=News).

The pYES-DEST52 cDNA expression library plasmids were separated using the SDS alkaline lysis method and then introduced into yeast strains via a polyethylene glycol (PEG)–lithium acetate-based transformation protocol [70] to screen the candidate stress-related functional genes. For the salt-sensitive mutant AXT3, the transformation products were plated on SDG-Ura solid medium (supplied with adenine) with 75 mM NaCl lacking uracil. The plates were placed at 30 °C for 3 to 7 days until single clones appeared. The plasmids from the surviving yeast clones were rescued in *E. coli* and extracted. About 100 plasmids in total were selected and sequenced with an automatic sequencing machine (ABI, Columbia, MD, USA). The plasmids containing the candidate cDNAs were also introduced into different yeast stains to perform the function verification, based on the same transformation protocol [70]. The basal SDG (Synthetic Dropout-/Galactose) medium was made with 1.7 g L^−1^ Yeast Nitrogen Base (YNB powder, BD Difco™, Bergen County, NJ, USA), 5 g L^−1^ ammonium sulfate, and 20 g L^−1^ galactose, with a pH value of 7.5.

### 4.5. Salt and H_2_O_2_ Sensitivity Assays in Yeast Cells

Following sequence confirmation of the plasmids isolated from the surviving yeast clones, the plasmids containing *I. pes-caprae* cDNAs were then transformed into yeast cells following Gietz and Woods to confirm the salt (W303 and AXT3) and H_2_O_2_ (WT, *yap1Δ*, and *skn7Δ*) tolerance of the candidate functional genes isolated from the cDNA library. In general, yeast transformants were precultured in SDG-Ura medium overnight at 30 °C, diluted with fresh prewarmed SDG-Ura medium, and then incubated with vigorous shaking for about 48 h at 30 °C to reach an optical density of just over 2.0 at OD600. Then, the cells were serially diluted in 10-fold steps, and 2 μL aliquots of each were finally spotted onto SDG-Ura agar medium with or without NaCl or H_2_O_2_. The NaCl concentrations used were 50, 75, and 200 mM for salt tolerance with the AXT3 yeast strain, or 0.85 M (mass-to-volume 5%), 1.28 M (mass-to-volume 7.5%), and 1.5 M (mass-to-volume 8.8%) NaCl separately for salt tolerance with the wild-type yeast strain W303. The H_2_O_2_ concentrations were 0.25 and 0.5 mM for the antioxidative assay with yeast strain WT (wild-type BY4741), *yap1Δ*, and *skn7Δ*. The test plates were incubated at 30 °C for 3 to 7 days.

### 4.6. Quantitative Reverse Transcription (qRT)-PCR Analysis

According to the sequences of the cDNAs screened from the cDNA library of *I. pes-caprae*, the qRT-PCR primer pairs were designed using the online program for Primer-BLAST with the following parameters: Tm of approximately 60 °C and product size range of 90–250 bp (available online: https://www.ncbi.nlm.nih.gov/tools/primer-blast/index.cgi?LINK_LOC=BlastHome). The partial cDNA sequence of the reference gene (*IpUBQ*) was cloned from *I. pes-caprae* and submitted to GenBank (accession number: MF502417), and was used as an internal control to normalize the amount of total cDNA present in each reaction. The primer sequences used for qRT-PCR analysis are listed in Supplementary Data Table S4. Generally, total RNAs were isolated with the HiPure Plant RNA Kit (Magen, Guangzhou, China) from the leaves, vines, and roots of *I. pes-caprae* seedlings according to the manufacturer’s instructions. Three parallel experiments were carried out to enable the assessment of significance. After assessing the concentration using a NanoDrop 2000 (Thermo Fisher Scientific Inc., USA), first strand cDNAs were synthesized by adopting TransScript One-Step gDNA Removal and cDNA Synthesis SuperMix (TransGen Biotech, Beijing, China) from 1 μg total RNA according to the manufacturer’s instructions. RT-PCR was conducted using a model 7500 Real-Time PCR system (Applied Biosystems, Thermo Fisher Scientific Inc., USA) and TransStart Tip Green qPCR SuperMix (TransGen Biotech, Beijing, China).

### 4.7. Statistical Analysis

All experiments were repeated at least three times in independent experiments, and the plant samples of independent experiments were harvested from at least five seedlings per treatment. The data analysis was performed using the statistical tools (Student’s *t*-test) of Excel 2010 (Microsoft, Seattle, WA, USA).

## Figures and Tables

**Table 1:** Annotation of 38 full-length *IpSRs* obtained from the yeast functional screening assay.

Clone	GenBank Accession No.	Functional Annotation	Length of cDNAs and Proteins Encoded by the Longest ORFs
IpSR1 (*IpASR*)	MF680587	putative ripening protein, abscisic acid, stress, and ripening-induced protein (ASR)	962 bp, 215 aa
IpSR2 (*IpATPD*)	MF680588	ATP synthase delta chain, chloroplastic	921 bp, 250 aa
IpSR3 (*IpNUD*)	MF680589	nudix hydrolase, chloroplastic	769 bp, 168 aa
IpSR4 (*IpTSJT1-1*)	MF680590	stem-specific protein TSJT1-like	1260 bp, 251 aa
IpSR5 (*IpCDI*)	MF680591	contact-dependent growth inhibition (CDI)-like protein	1262 bp, 277 aa
IpSR6 (*IpFBP*)	MF680592	F-box protein At5g46170-like	1538 bp, 388 aa
IpSR7 (*IpCAB21*)	MF680593	chlorophyll a-b binding protein 21, chloroplastic	1013 bp, 267 aa
IpSR8 (*IpERVT*)	MF680594	endoplasmic reticulum vesicle transporter, C-terminal	1428 bp, 386 aa
IpSR9 (*IpTSJT1-2*)	MF680595	stem-specific protein TSJT1-like	1196 bp, 237 aa
IpSR10 (*IpPI1*)	MF680596	probable proteasome inhibitor	1115 bp, 343 aa
IpSR11 (*IpFRK*)	MF680597	fructokinase	1312 bp, 324 aa
IpSR12 (*IpPABP*)	MF680598	polyadenylate-binding protein RBP47B′ isoform X1	1598 bp, 423 aa
IpSR13 (*IpTC*)	MF680599	probable tocopherol cyclase, chloroplastic	2007 bp, 487 aa
IpSR14 (*IpSNARE*)	MF680600	hypothetical protein, Syntaxin/t-SNARE family protein	1743 bp, 346 aa
IpSR15 (*IpPT*)	MF680601	probable inorganic phosphate transporter 1-3	1918 bp, 540 aa
IpSR16 (*IpAHCY*)	MF680602	adenosylhomocysteinase 1	1844 bp, 485 aa
IpSR17 (*IpPUP*)	MF680603	peptide upstream protein	2123 bp, 453 aa
IpSR18 (*IpCAT*)	MF680604	catalase	1758 bp, 492 aa
IpSR19 (*IpSRP*)	MF680605	stress responsive alpha-beta barrel domain protein	847 bp, 221 aa
IpSR20 (*IpRPB7*)	MF680606	DNA-directed RNA polymerase II subunit RPB7	976 bp, 177 aa
IpSR21 (*IpUSP3*)	MF680607	ubiquitin carboxyl-terminal hydrolase 3	1536 bp, 368 aa
IpSR22 (*IpRPS25*)	MF680608	40S ribosomal protein S25	557 bp, 108 aa
IpSR23 (*IpPI2*)	MF680609	probable proteasome inhibitor	1116 bp, 300 aa
IpSR24 (*IpHP1*)	MF680610	hypothetical protein AT3G52710	997 bp, 225 aa
IpSR25 (*IpPMM*)	MF680611	phosphomannomutase	1048 bp, 246 aa
IpSR26 (*IpLEA*)	MF680612	desiccation-related protein At2g46140	1392 bp, 313 aa
IpSR27 (*IpLRRK*)	MF680613	protein kinase superfamily protein	1732 bp, 385 aa
IpSR28 (*IpDNAJ*)	MF765747	dnaj protein-like protein	1595 bp, 428 aa
IpSR29 (*IpPSK*)	MF680614	phytosulfokines-like	721 bp, 81 aa
IpSR30 (*IpPSCXI*)	MF680615	photosystem I reaction center subunit XI, chloroplastic	803 bp, 218 aa
IpSR31 (*IpSCP*)	MF680616	sugar carrier protein C	1877 bp, 528 aa
IpSR32 (*IpHRGP*)	MF680617	hydroxyproline-rich glycoprotein family protein	930 bp, 215 aa
IpSR33 (*IpGST*)	MF680618	glutathione S-transferase L3-like isoform X1	1022 bp, 234 aa
IpSR34 (*IpABAH*)	MF680619	abscisic acid 8′-hydroxylase 4	1748 bp, 465 aa
IpSR35 (*IpSR45a*)	MF680620	serine/arginine-rich splicing factor SR45a isoform X2	1106 bp, 243 aa
IpSR36 (*IpFMT*)	MF680621	quercetin 3-O-methyltransferase 1	1277 bp, 356 aa
IpSR37 (*IpGBP*)	MF680622	guanine nucleotide-binding protein subunit beta-like protein	1253 bp, 326 aa
IpSR38 (*IpDHN*)	KX426069	dehydrin	983 bp, 217 aa

## Supplementary Materials

Supplementary materials can be found at http://www.mdpi.com/1422-0067/19/11/3446/s1.

## Abbreviations

FOX	Full-length cDNA Over-eXpressor
SR	Salt-stress Related
qRT-PCR	Quantitative Reverse Transcript-Polymerase Chain Reaction
ROS	Reactive Oxygen Species
SCBG	South China Botanical Garden
NCBI	National Center for Biotechnology Information
RIN	RNA Integrity Number
ORF	Open Reading Frame
LB	Luria-Bertani
MS	Murashige and Skoog
SDG	Synthetic Dropout/Galactose
OX	Over eXpression
