# Supplementary material for: Functional Identification of Salt-Stress-Related Genes Using the FOX Hunting System from Ipomoea pes-caprae
Source: Int J Mol Sci. 2018 Nov 2;19(11):3446. doi: 10.3390/ijms19113446 (PMC6274920; doi:10.3390/ijms19113446)
Supplement: Supplementary file 1 [file ijms-19-03446-s001.zip › ijms-342421-supplementary-11-1.pdf]

Supplementary Information

Table S1. Sequence analysis of 42 randomly selected clones (*Ipseq1-Ipseq42*) from the cDNA expression library of *I. pes-caprae*.

Table S2. 5′ UTR sequence (for the longest ORFs) analysis of 42 randomly selected clones in the pYES-DEST52 cDNA expression library of *I. pes-caprae*.

Table S3. The sequence information of *I. pes-caprae* cDNAs listed in this paper.

Table S4. Primers for RT-PCR detection of candidate salt stress related genes responding to NaCl and mannitol.

Figure S1. RT-PCR analysis of the other 31 salinity-tolerant candidate genes (*IpSRs*) and *IpUBQ* in the roots, vines, and leaves of *I. pes-caprae* seedlings under salinity (300 mM NaCl) stress at 0 and 24 h. All determinations were carried out for three biological replicates.

Figure S2. RT-PCR analysis of the other 31 salinity-tolerant candidate genes (*IpSRs*) and *IpUBQ* in the roots, vines, and leaves of *I. pes-caprae* seedlings under dehydration (right, 300 mM mannitol) at 0 and 24 h. All determinations were carried out for three biological replicates.

Table S1

Clone	GenBank accession number	Functional annotation	Length of cDNAs and proteins encoded by the longest ORFs	Note
Ipseq1	MF680552	L-arabinokinase-like isoform X1	1092 bp, 311 aa	Partial
Ipseq2	MF765740	uncharacterized protein At1g04910-like	1876 bp, 513 aa	Complete
Ipseq3	MF765741	21 kDa protein-like isoform X1	822 bp, 202 aa	Complete
Ipseq4	MF680553	zinc finger CCCH domain-containing protein 29-like	1446 bp, 374 aa	Partial
Ipseq5	MF680554	quinone oxidoreductase	1158 bp, 193 aa	Partial
Ipseq6	MF680555	uricase-2 isozyme 1	1092 bp, 244 aa	Partial
Ipseq7	MF680556	60S ribosomal protein L24	941 bp, 124 aa	Partial
Ipseq8	MF680557	zinc finger CCCH domain-containing protein 29-like	916 bp, 169 aa	Partial
Ipseq9	MF680558	pyruvate dehydrogenase E1 component subunit beta-1, mitochondrial-like isoform X2	1500 bp, 231 aa	Complete
Ipseq10	MF680559	uncharacterized protein	961 bp, 185 aa	Partial
Ipseq11	MF765742	V-type proton ATPase subunit C	1515 bp, 376 aa	Complete
Ipseq12	MF680560	JAZ1	1531 bp, 104 aa	Complete
Ipseq13	MF765743	70 kDa peptidyl-prolyl isomerase-like	1208 bp, 312 aa	Partial
Ipseq14	MF680561	hypothetical protein MANES_03G205700	1492 bp, 410 aa	Complete
Ipseq15	MF680562	thiosulfate sulfurtransferase 16, chloroplastic-like	925 bp, 184 aa	Partial
Ipseq16	MF680563	glucan endo-1,3-beta-glucosidase 13-like isoform X2	963 bp, 225 aa	Partial
Ipseq17	MF765744	alanine aminotransferase 2	1741 bp, 483 aa	Partial
Ipseq18	MF680564	uncharacterized protein LOC104246255 isoform X5	943 bp, 90 aa	Partial
Ipseq19	MF680565	UDP-glycosyltransferase 91A1-like	1129 bp, 233 aa	Partial
Ipseq20	MF680566	probable glycosyltransferase At5g03795 isoform X1	712 bp, 146 aa	Partial
Ipseq21	MF680567	cathepsin B-like cysteine proteinase	1250 bp, 353 aa	Complete
Ipseq22	MF680568	protein EXORDIUM-like precursor	1120 bp, 328 aa	Complete
Ipseq23	MF680569	DNA-directed RNA polymerases II, IV and V subunit 6A-like	666 bp, 143 aa	Complete
Ipseq24	MF680570	peroxisome biogenesis factor 10	1982 bp, 401 aa	Complete
Ipseq25	MF680571	Uncharacterized protein TCM_029159	957 bp, 180 aa	Complete
Ipseq26	MF680572	WD40 repeat protein	1277 bp, 343 aa	Complete
Ipseq27	MF680573	profilin-4	742 bp, 131 aa	Complete
Ipseq28	MF680574	serine/threonine-protein kinase HT1-like	1837 bp, 412 aa	Complete
Ipseq29	MF680575	UDP-D-apiose/UDP-D-xylose synthase 2	1557 bp, 386 aa	Complete
Ipseq30	MF680576	PDR1-like protein	1059 bp, 219 aa	Partial
Ipseq31	MF680577	wound-induced protein 1	655 bp, 107 aa	Complete
Ipseq32	MF680578	glyceraldehyde-3-phosphate dehydrogenase C	1355 bp, 338 aa	Complete
Ipseq33	MF680579	cation transport regulator-like protein 2	1319 bp, 226 aa	Complete
Ipseq34	MF680580	ribosomal RNA-processing protein 14-C	1272 bp, 123 aa	Partial
Ipseq35	MF680581	acetyl-CoA acetyltransferase, cytosolic 1 isoform X2	1218 bp, 289 aa	Partial
Ipseq36	MF680582	probable galacturonosyltransferase-like 10	1254 bp, 366 aa	Complete
Ipseq37	MF680583	xyloglucan endotransglucosylase XET1	1049 bp, 296 aa	Complete
Ipseq38	MF765745	isocitrate lyase-like	2045 bp, 575 aa	Complete
Ipseq39	MF680584	WW domain-containing protein C11B10.08	1177 bp, 267 aa	Complete
Ipseq40	MF680585	probable E3 ubiquitin-protein ligase XERICO	830 bp, 195 aa	Complete
Ipseq41	MF680586	asparagine synthase (glutamine- hydrolyzing)	1782 bp, 473 aa	Partial
Ipseq42	MF765746	LOX3	2723 bp, 829 aa	Partial

Table S2

Clone	Upstream sequence of Start codon ATG
Ipseq1	AGGTAGAAAACCATGTTGTTGGGGCTCCATGTGGTGTA**ATG**GAC (-38 to +6)
Ipseq2	ACCCAGTGCCGACCACC**ATG**TGG (-17 to +6)
Ipseq3	AG**ATG**AAA (-2 to +6)
Ipseq4	CTCAGTACAAGACCCGCCTGTGTAAGGATGAGACTGGATGCTCGAGGAAAGTGTGCTTCTTTGCT<br>CACAAGCCCGAGGAGCTACGCCCACTGTATGCTTCAACTGGCTCGGCCATTCCTTCGCCAAAGTC<br>TGGTTCAGTGAGTTCA**ATG**GAC (-146 to +6)
Ipseq5	GCAGTTGTGATCACAACGCCCGGCGGACCGGAAGTCCTGCAAATTCAAGAAGTGGAAGACCCGA<br>AGATCAAGGACGATGAAGTGCTGATCAGAGTGGCAGCCACCGCGCTGAACAGAGCCGACACCCT<br>TCAGCGCCAAGGAAAATACCCACCGCCGAAGGGCGACAGTGAATACCCAGGTCTTGAGTGCTCC<br>GGCACCGTTGAAGCTGTGGGCAAAAATGTCGAAAAGTGGAAAGTGGGTGACCAGGTGTGTGCTC<br>TCGTTGGTGGTGGAGGCTATGCTGAGAAAGTAGCTGTACCTGCTGGACAGGTTCTTCCTGTTCCAT<br>CAGGTGTTTCTTTGCAAGATGCAGCTAGTTTCCCAGAGGTGGCGTGTACTGTTTGGTCGACTGTCTT<br>T**ATG**ATG (-390 to +6)
Ipseq6	AGGACGGATTTAAGCTGCAGCAGAGGCACGGGAAGGCAAGGGTGAGAGTTGGCCGGGTATGGA<br>GAGACGGAGACGGTACGCATCACTTTTCCGAATGGAACGTCAGCATAAGCCTCCTCTCCGACTGC<br>CTCCCCGCCTACGTCGCAGGCGACAACTCAGACATCGTCGCCACCGATACC**ATG**AAG (-179 to +6)
Ipseq7	CAACACAGTTCTCAGCAGCC**ATG**GTT (-20 to +6)
Ipseq8	TTCCTTGTCCCATTTGCAGGGTCTCTCGCCAAAGGTTGCAAGAAGCAGCAGCTCCCAGCTGCAAT<br>CCCCAACCGGGCTTCAAATTCGCCAAAAC**ATG**AAC (-94 to +6)
Ipseq9	GAATCTGCTGCGCTTGCCATAACAAATACGAAGCCGGCTTTTAGGAATTTTGTTGCCCAAACATTC<br>CTCCATCAAACAGTCAAATATCTCCCAGGGCTGTGATTGCGTCAGTAGAAGAGGATTGAT**ATG**TCT<br>(-126 to +6)
Ipseq10	ACAGAATAATGCCGAACAAGAAATATCTGATACCAGTAAATCAGACCCAATTACAGAAGAGCAAT<br>CAGAGGACACTCAG**ATG**GTA (-79 to +6)
Ipseq11	ACGAGAATACAGAGTGAGTGATCAGAGAGGTGTCGAACTGGTTTGATCCATTTCTATTTCCTGCAA<br>TCCAACTCCAATTGTTGCAGTAAAGCACGAAGAAGAATAAGAGATCCGATTCCA**ATG**GCG (-120 to +6)
Ipseq12	GAAATCTGATATGCCTAATGCTGCAACCACTATCGACTTCCTATCCAACATGGAGAAATCAAGTCA<br>GACTCCAGAACTGGACTTTAATTCGACAGATCCCCTCCCTGAGCAGGATGATGCTATGGGGTCTGT<br>TAGAACAATGGAGGAAAACGCAAATGAAGCTAGTATTAGGTTTTTCCCATGTTTTACACCTTCATT<br>GTTTCATACAAATAATGCTCAGTTCATACATTTCTTGCTTGTCTTGTTTTGAGAGATGGTTCTGATAT<br>C**ATG**TGT (-267 to +6)
Ipseq13	AAGATGGAACTATTTTTGTTAAAAAGGGGAGCGATGAGGAACCATTTGAATTCAAAGTTGATGAA<br>GACCAAGTTATTGATGGACTTGACAAAGCTGTAAAAACA**ATG**AAG (-104 to +6)
Ipseq14	TTATTTTATTTAGTGTTTTTAATTTTATTTTTTCCCCCATTTTGGAGCTCCATGTGGG**ATG**CTC (-58 to +6)
Ipseq15	AAAGTTATCTCTGCCCCAACGTACAACTCTATAGTGAACCTATCAACCTAGCTAAGATCATCGTATT<br>CATCTGAACTGAAGCGGCCGCAGCA**ATG**ATG (-92 to +6)
Ipseq16	GATCCTAACAATGGCCTTGTTTACAAAAGCCTGCTGGAGGCGCAACTCGACGCCGTCTTCGCCGC<br>C**ATG**AAA (-66 to +6)
Ipseq17	TATTCGTCCACTCCCCTCACTCTTCAATCCATCAATCCCAAGGTTCTGAAATGTGAGTATGCTGTTC<br>GTGGAGAAATTGTCAGCCTTGCTCAGAAAATACAAGAAGAGTTACAGGCAAAACCAGGTTCTCA<br>CCCATTTGATGAGATAATTTATTGCAATATTGGAAACCCTCAATCCCTTGGTCAGCAGCCAATTACT<br>TTCTTTAGGGAGGTGCTTGCATTATGTGACCATCCTTCTATTTTGGATAAAAGTGAAACTCAAGGCC<br>TCTTCAGTGCGGACTCCATAGAGCGAGCTTTCCAGATCCTTGATCAAATTCCAGGAAGAGCAACT<br>GGTGCATACAGCCATAGTCAGGGTATCAAAGGATTGCGTGATACAATTGCTTCTGGTATAGAAGCT<br>CGCGATGGTTTCCCTGCCAATCCAAATGACATTTTCTTGACTGATGGTGCAAGCCCAGCGGTTCAC<br>**ATG**ATG (-462 to +6)
Ipseq18	ATTCTAAAGTGCACAGGAGGTTCTATACAGTAAACGCCAATGGATGCAACAACAATAGTACTGCT<br>CCCCCGTGTGTAGGGTGGGTAGAA**ATG**ATG (-89 to +6)
Ipseq19	AGGAAGAAGATAACGTTTCCGATGTTTTCCGA**ATG**CTT (-32 to +6)
Ipseq20	CTACTCCGGCGGCCTCCACGGCCCCATACGCCCAATCCTG**ATG**CAA (-40 to +6)
Ipseq21	AGAAACAAGAAAAAAA**ATG**GAG (-15 to +6)
Ipseq22	CATTTCTCTCTTCTGATCTCTGATATTTTAGTGAAAAATCCCAGA**ATG**GCT (-45 to +6)
Ipseq23	GTTTCGGAGCTTTGCTGGTAGGGAGAAGCACCTCAAAA**ATG**GCG (-38 to +6)
Ipseq24	TCGCCCTCTCGGTCTCTGCCTCTGCCCGGACTGCCCCGTCGCTCGCCAGTCGCCACGGCCTACGAG<br>ACAGTGAGACACCGTCACCGGTCACACCAGACAACCAGTCGCCGCTTCGCCACTCGCCAGCTTC<br>ATCTTCAAGCTTCAACCGTCTTCACCAGTTCACCCTCAGCTCCGTGCCTCCGTGACTCCGTCCCTG<br>CCGACTGCCGTCAGTCCATCTTCACCCTCAGGCCTCAGTTCTGATTCCGTCCCTGGGCTGAGAG**AT**<br>**G**GGA (-260 to +6)

Ipseq25	TCATATGAATTTTATCTCTTTATCTTTTGCTTATTACTATGAGATCTCAATGAGATTTATGTAACGGAA<br>AGGAAGAGTTTGTGTATTTAATTGAGAAATATGTGTTCTATGGATTCATTTTTGTGCCGGACGATGC<br>AAAGGCGGAGGAAGGCGGGCGGGGGTCGGGAAGGTAGTGATTGAAGATTGAGTTTGCTGAGTAT<br>CCTCTGTGGAGAAGCAGAAGCAGAGATAAATTTTCTGTGGACAGTAATAATTTGTTGGAGGTTTTT<br>ATTCTGTCTGTTTAATCTCGGCTTCTGCTGCT**ATG**TCG (-298 to +6)
Ipseq26	GAGGGAAGATCAGATTTTAACCCATTTTCCTTCAAAG**ATG**GAG (-37 to +6)
Ipseq27	TCTCTCTCTCTACACACAACATAGCGCAGCAATTGTAGCCGGAAAATTCTCATCGGAGAATTTCGA<br>GG**ATG**TCG (-68 to +6)
Ipseq28	CACGCATTCATTCAATTGCTCGGCTTGCCTATCTATTTCTCTCTCTATTTCTCGGACTGCTACCTCTCT<br>CTCTAGCATCCTCGCCGGTTCGGTGCAGCAGCCGTGATAATTCAACTGAGGTGGAAGTGGCGTGC<br>GGTGGTTGGGTAAACGTCATCGTTTGGGATATCGATCGGCGTGTGAACTGAAAAAGGTAACTAAG<br>CCGATCCATAGACCTTTAGTACACAGAATTTAAGGCATATGCAAAGTTAGATTTGAGGAATTAAAT<br>GCTCATGCAGAAGAATAAGTCAAGGTAAAATAAATATAATTTTTTTGTGCTGCAGCAGTGGTGTCA<br>GAGAAG**ATG**TTG (-337 to +6)
Ipseq29	-AAATCTAGAGAGGTGAGTGAGTCAGAACTTAGAAGGCAAAAGTCTGATATACATACGTACAGGA<br>ATATCAGCGTTCGTGAAGGTGAGAGGAGAAGATAATTGCAGAGCTTGTGTCGACGGAA**ATG**GCC<br>(122 to +6)
Ipseq30	CACTTCCCTACCAAGTTCTCACAAAGTTTCTTCAGCCAATGTTTGGCTTGCTTCTGGAAGCAACGC<br>CTGTCCTACTGGCGTAACCCGGCTTACACCGCAGTTAGATTCTTCTTCACAACCTTCATCGCTCTCA<br>CATTCGGAACCATTTTCTGGAAACTCGGCAAGAAAACTGAAAAGAGACAAGATCTGATTAACGCT<br>**ATG**GGA (-198 to +6)
Ipseq31	CTTCTTCCAACATCAACACCACACTAGCCTCCCCCTCTCTCTCTCTCTCTCTCTCTGTATCTATCT<br>CTTTCTCTCTCTATAAATATATATATATATCTGTCTTCCGGTCTGGCTAACATTCAGGAAACTCTGGA<br>GGAAACCAGAAACAAGAGGGTGGTGCTCGCCCTGTATGAAGCCTTGAGCTCCCGAGACGTGGAT<br>TTGGTCCAGAAACTTCTGGCCTCTGACCTAGAGTGGTGGTTCCATGGTCCGCCGTCCCACCAGTTT<br>TTG**ATG**CGC (-269 to +6)
Ipseq32	CAACTCTTTCTAAACCCTCAAAACCATTCTCTCAGTCTCTCTCTCTCTCTCTCTCTCAAACTCCGCC<br>**ATG**GCG (-67 to +6)
Ipseq33	GAGAGGCGAGGTTCAAGGAGAGAATAAAGCCAAGAAGATCACAGGCCTCTCTCTCTCTCTCTC<br>TCTCCCCTAGGGTTTGCAGTTTCCATTGATTCCCATTTTGCTTTGTACTCTGCCCAATCTTATTCAGA<br>GAACTATATTTGTTGGAAAAAGAAAGGATTTTGTTGTTTCAGTTGATTCAGAAGCCTTCTTGGTGGA<br>TATTGTTTTTTTGATTGTGGAAGAAGGTTGATCAA**ATG**GTG (-235 to +6)
Ipseq34	GAAGTCCGTTGCCGTCGACGCGGCGGCCATCGCTGTCGCAGCTACTGACCTGAAAGCACTTATTC<br>ACGAGAGCAGCGTCTTCTTTGACAAGTTGGTAAATCTCATCCCACCCAGATTCTACCTACCAAGCG<br>AAGACTCCAAGCCATGGTTTCAAGGCTTGTCCAAAGCCAAAAAGGCCTCTTTCAAACGACAGTC<br>CAGGGAGAACCTCAAACTCGCCCGCCGTAATCGCCTCGACCCGGAGAAGAAGATTGCCCAATCC<br>TCAACTCTCGACCTCCTCAAAGAGTCCATCGAGAAGCAGAAAATTGCGGAACTATCGGACGATTC<br>TGACAAAGATAAACCTATCGGTTTCGGCGAAGATGATGGTAACGAGAATGCTGATGATCGATCGG<br>TTACCTACGAAGAGTTGCGGCAGAGGCTCCGGAGGAAAATCGAAGCCCTTCGTGGTAACCGAGG<br>GGCTGGTGAGAAATCAGAGCGGAAAGGAAGTGGCAAAGATGGAAATTCGGTGAACTCAGAAGG<br>GAAGAAGCGAAAGAGGAATAGTGACGGAGGTGGACATAGTGAAAAGACGGGCAATGAGGCAAA<br>TCCGGGGAATGTTCTGAGTGAAATTGAATTTGGGAAAGTAAAGATTGGGGACGATGACAAGCAG<br>GGGAAGAAGAAGAAGAAGAAGAAG**ATG**TCA (t-667 o +6)
Ipseq35	GCACTTAGCATTACATCGGGTGTCAATGATGTTGTGGTGGCTGGCGGG**ATG**GAA (-48 to +6)
Ipseq36	AAATCTCTGGCTTTTC**ATG**TTA (-16 to +6)
Ipseq37	ATTCCCTTTCCCAAAATCACCAGAAAGCTAAGAT**ATG**AAG (-34 to +6)
Ipseq38	CTCACCATTTACTGCACCTTCAATGATT**ATG**GAA (-28 to +6)
Ipseq39	CTTTTGTAGTCTTGAGATAATTTTGAGGGCATCAATAATATCATTGTTTGTTTCGCCGGAAAGCGTTG<br>GGA**ATG**GGA (-71 to +6)
Ipseq40	TAAAATTAAACAAGAA**ATG**AGA (-16 to +6)
Ipseq41	CATTGCGCATCTTTATGAAGAACATGGAGAGAACTTCGTGGAC**ATG**CTG (-43 to +6)
Ipseq42	TGTGGTGACTGTGAGGAACAAGGACAATGAAGATTTTAAGGAAACTTTTGTCAAGCATCTTGATA<br>ATCTCTCTGAAAATTTTGGCAGAAATGTTGTG**ATG**GAG (-97 to +6)

Table S3

Supplementary table 3.xlsx

Table S4

Clones/Genes	Sequences of primer pairs (RT-F/RT-R, 5′-3′)	Amplicons
IpSR1 (*IpASR*)	AAGATAGCAGAAGAGGTGGCG/ACGCATAAGCCCAAACCCAT	234 bp
IpSR2 (*IpATPD*)	ACATCGTGCAGGAGTTCGAG/GAATCGCCATACCGGATCGT	205 bp
IpSR3 (*IpNUD*)	AGAGGTACAGCGACAACACC/TTCTGCGAGCTGATCACGAG	106 bp
IpSR4 (*IpTSJT1-1*)	GTGTTCAACAAGTCGGTGGC/CCAAGGTTGATGGTGACGGA	140 bp
IpSR5 (*IpCDI*)	TCCCCATTGAAATCCACCCG/AAACATGGCCCAACCCTCAT	149 bp
IpSR6 (*IpFBP*)	TGACTGCGTTATCTCCGACG/GTCACGGAGCTCTGGTCAAA	237 bp
IpSR7 (*IpCAB21*)	CTTCCACCCCGGAAACTACC/CTTAACACGGTCTGGGCCAT	110 bp
IpSR8 (*IpERVT*)	CCTTTACGGAGGAGCACGTT/CGCTTTTTGGCCGTGGTAAA	119 bp
IpSR9 (*IpTSJT1-2*)	GGTCACCTTGAGGGCAACTT/GAAGAAGCACCCTTGTGGGA	201 bp
IpSR10 (*IpPI1*)	TCCATTTGGTCCACCTGACG/GCTCCAGATCAGGGTGGTTC	97 bp
IpSR11 (*IpFRK*)	GACCCCGGAGGAGTTGAATC/CCTCAGGTGATGGCCACAAT	185 bp
IpSR12 (*IpPABP*)	CCATTACTCCGACTCCGACG/GAGATAAGGCTCTTCGGCCC	200 bp
IpSR13 (*IpTC*)	TGCCAGGCCTCCAAATAAGG/GCGAGCAGTTTTCACGGTTT	145 bp
IpSR14 (*IpSNARE*)	TGTGCGCATCTCAATCCTCT/CCAGCATCTTTGCTTCTCGC	108 bp
IpSR15 (*IpPT*)	GCGACTATCCACTCTCTGCC/CGTAAGCGTGATCGAAAGCG	156 bp
IpSR16 (*IpAHCY*)	CAAGGCCGATCCCAAGAAGT/TGGCGGCATCCATACAAGTT	183 bp
IpSR17 (*IpPUP*)	TCGCTGGTGGGGAGATTAGA/TTGGCTTTTCACCAACCCCT	105 bp
IpSR18 (*IpCAT*)	GGCCCCAACTATATGCTGCT/ACGTAACGGAGTTGGGAACC	165 bp
IpSR19 (*IpSRP*)	GGGGGATTGGATGCTGTGAA/GAACAGACTCTTCGCCGGAA	129 bp
IpSR20 (*IpRPB7*)	AAGACGTCGAGGGAACTTGC/GGGAAAGTGACGAATCCCGT	112 bp
IpSR21 (*IpUSP3*)	AACCACGGGCACTATGTCAG/TGGTGCCAGAACATACGCTT	181 bp
IpSR22 (*IpRPS25*)	GAACAACCAGAATGGCTCCG/AGCTTGTCATAGGTGGCCTT	163 bp
IpSR23 (*IpPI2*)	ATGGCATGCTTGTAGGACCC/AACGACCTGGCTCAAAACCA	150 bp
IpSR24 (*IpHP1*)	TGTCGGCGAAGAAGACGAAA/TGCTCGGAGTCTTTGGGAAC	129 bp
IpSR25 (*IpPMM*)	TCGCACACCTTGATCTCACC/TACACTGCTTTGCCGTGTCT	225 bp
IpSR26 (*IpLEA*)	ACCAACTGCAGATGTGTCCG/CGTTCCAGCATCAGGGATCA	181 bp
IpSR27 (*IpLRRK*)	CTGAGTATGCAATGCGTGCC/GTAGGCGCTTGTTGGTGTTG	108 bp
IpSR28 (*IpDNAJ*)	ACAGATTGCACCTGGCATGA/CCCCTTCTCGACATGCACTT	157 bp
IpSR29 (*IpPSK*)	TTTCATCGTAGCGCTCCTCC/CAGAGTCCTCCGCATCAGAC	172 bp
IpSR30 (*IpPSCXI*)	CCCATAACGTCAAGCCCACT/CACAAATGGACCCACCAGGA	132 bp
IpSR31 (*IpSCP*)	CATCGGCCGTATTTTGCTGG/ACGCCATCCCCAATGGATTT	190 bp
IpSR32 (*IpHRGP*)	GACGACGACGACTCAGATGG/TTGGGTGATGATATCCGCCG	140 bp
IpSR33 (*IpGST*)	TCCAGTGCCCTTATGCTCAG/TTGTTGTGCTCCAATGCAGG	157 bp
IpSR34 (*IpABAH*)	CATGCCATTTGGCAGTGGAG/CGGGAATGGGCCATACTGAA	145 bp
IpSR35 (*IpSR45a*)	GATGCCAATCGCTGCGTAAA/ACGATAACCTTCTGCCCTGC	147 bp
IpSR36 (*IpFMT*)	GTGACGGACAAGGAAGGGTT/TCCGGCTTCTTCCCGTAGTA	170 bp
IpSR37 (*IpGBP*)	GTGCTGTCCGTTGCTTTCTC/ACCCATTCGGTGTGACCATC	134 bp
IpSR38 (*IpDHN*)	CCTGGGTACCACCCAAAGAC/TAAAGTACTTACAGCAAACC	246 bp
IpUBQ	TCGACAATGTGAAGGCAAAG/CTTGATCTTCTTCGGCTTGG	209 bp

Figure S1

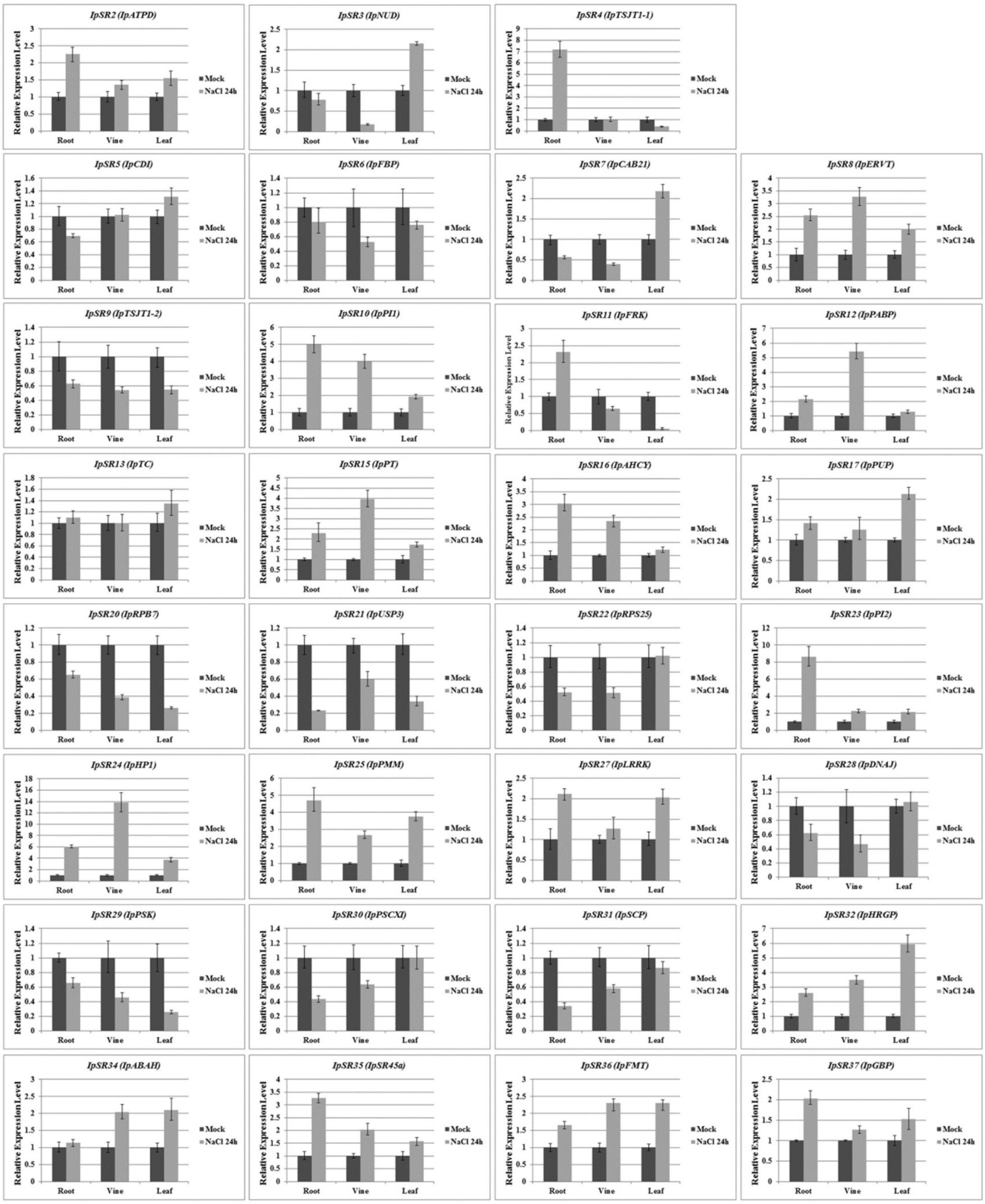
IpSR2 (IpATPD)
IpSR3 (IpNUD)
IpSR4 (IpTSJT1-1)
IpSR5 (IpCDI)
IpSR6 (IpFBP)
IpSR7 (IpCAB21)
IpSR8 (IpERVT)
IpSR9 (IpTSJT1-2)
IpSR10 (IpPI1)
IpSR11 (IpFRK)
IpSR12 (IpPABP)
IpSR13 (IpTC)
IpSR15 (IpPT)
IpSR16 (IpAHCY)
IpSR17 (IpPUP)
IpSR20 (IpRPB7)
IpSR21 (IpUSP3)
IpSR22 (IpRPS25)
IpSR23 (IpPI2)
IpSR24 (IpHP1)
IpSR25 (IpPMM)
IpSR27 (IpLRRK)
IpSR28 (IpDNAJ)
IpSR29 (IpPSK)
IpSR30 (IpPSCXI)
IpSR31 (IpSCP)
IpSR32 (IpHRGP)
IpSR34 (IpABAH)
IpSR35 (IpSR45a)
IpSR36 (IpFMT)
IpSR37 (IpGBP)
Relative Expression Level
Root
Vine
Leaf
Mock
NaCl 24h

Figure S2

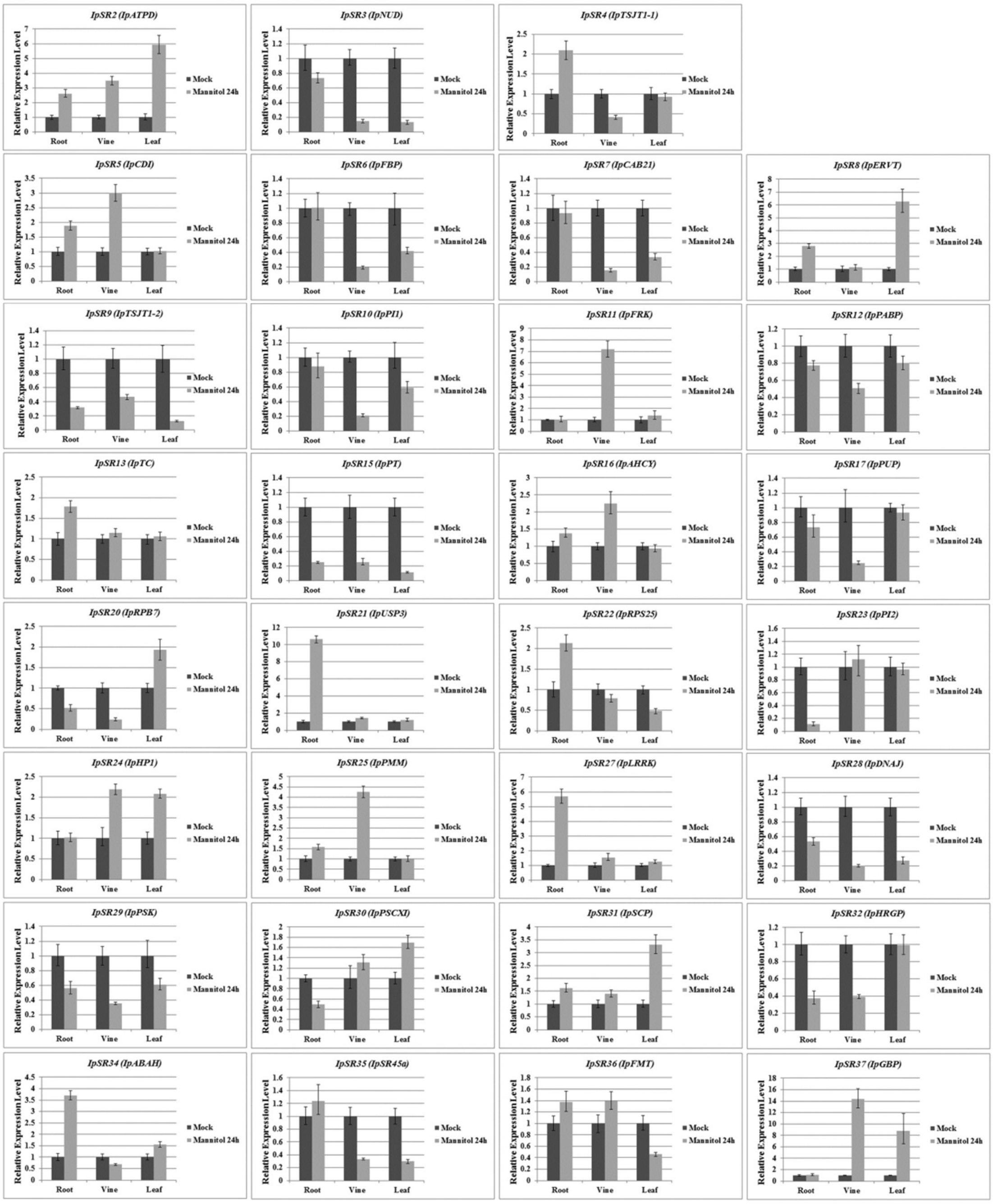
IpSR2 (IpATPD)
IpSR3 (IpNUD)
IpSR4 (IpTSJT1-1)
IpSR5 (IpCDI)
IpSR6 (IpFBP)
IpSR7 (IpCAB21)
IpSR8 (IpERVT)
IpSR9 (IpTSJT1-2)
IpSR10 (IpPI1)
IpSR11 (IpFRK)
IpSR12 (IpPABP)
IpSR13 (IpTC)
IpSR15 (IpPT)
IpSR16 (IpAHCY)
IpSR17 (IpPUP)
IpSR20 (IpRPB7)
IpSR21 (IpUSP3)
IpSR22 (IpRPS25)
IpSR23 (IpPI2)
IpSR24 (IpHP1)
IpSR25 (IpPMM)
IpSR27 (IpLRRK)
IpSR28 (IpDNAJ)
IpSR29 (IpPSK)
IpSR30 (IpPSCXI)
IpSR31 (IpSCP)
IpSR32 (IpHRGP)
IpSR34 (IpABAH)
IpSR35 (IpSR45a)
IpSR36 (IpFMT)
IpSR37 (IpGBP)
Relative Expression Level
Mock
Mannitol 24h
Root
Vine
Leaf
